# Association of the Endothelial Nitric Oxide Synthase Gene T786C Polymorphism with In-Stent Restenosis in Chinese Han Patients with Coronary Artery Disease Treated with Drug-Eluting Stent

**DOI:** 10.1371/journal.pone.0170964

**Published:** 2017-01-27

**Authors:** Wen-ping Zeng, Rui Zhang, Ran Li, Jin-fang Luo, Xiao-feng Hu

**Affiliations:** 1 Department of Cardiology, Zhejiang Hospital, Hangzhou, Zhejiang Province, China; 2 Department of Cardiology, Nanchang University Second Affiliated Hospital, Nanchang, Jiangxi Province, China; Children's National Health System, UNITED STATES

## Abstract

**Background and aim:**

Many studies have reported that genetic variants correlate with higher risk for coronary artery disease (CAD) or in-stent restenosis (ISR) after bare metal stent (BMS) implantation. However, there is limited data assessing the impact of these variants on ISR in patients treated with drug-eluting stent (DES). The purpose of this study was to investigate the effects of genetic risk factors on ISR in Chinese Han patients treated with DES.

**Methods:**

A total of 425 patients with a diagnosis of CAD who underwent successful revascularization in native coronary arteries with DES were included in this retrospective study. Genotyping was performed on six single nucleotide polymorphisms (SNPs) in the endothelial nitric oxide synthase gene (*eNOS)*, the angiotensin converting enzyme gene (*ACE)*, the angiotensin II type 1 receptor gene (*AT1R)*, the transforming growth factor beta gene (*TGF-β)*, and the vascular endothelial growth factor gene (*VEG*F). Quantitative coronary angiography (QCA) was performed during the follow-up period to detect ISR. Logistic regression models were used to test for association.

**Results:**

Fifty-four patients (12.7%) developed ISR during the follow-up period. Of the six analyzed SNPs, the frequency of the C allele of T786C polymorphism in *eNOS* was significantly higher in the ISR group (22.2%) compared to the non-ISR group (12.7%) (p<0.01). In the ISR group, the frequency of the TT, TC, and CC genotypes was 61.1%, 33.3%, and 5.6%, respectively, and in the non-ISR group, the frequencies were 76.8%, 21.0%, and 2.2%, respectively. The multivariable analysis adjusted for potential confounders and revealed that the T786C polymorphism increased the risk of ISR in both additive and dominant models with odds ratios of 1.870 (95% confidence interval [CI]: 1.079–3.240, p = 0.03) and 2.045 (95% CI: 1.056–3.958, p = 0.03), respectively.

**Conclusion:**

The *eNOS* T786C polymorphism was associated with ISR in Chinese Han patients treated with DES. Genotyping may be helpful to identify patients with higher risks of ISR after DES implantation.

## Introduction

Cardiovascular diseases, particularly coronary artery disease (CAD), remain the leading cause of mortality worldwide. Percutaneous coronary intervention is widely used for the treatment of coronary artery disease and results in better outcomes than balloon angioplasty alone [1]. However, after a successful intervention, in-stent restenosis (ISR) can develop, leading to the recurrence of myocardial ischemia symptoms that are not completely relieved by drug-eluting stents (DES) [2–4]. Thus, development of ISR in patients undergoing percutaneous coronary intervention (PCI) with DES remains a critical problem limiting successful treatment.

Multiple clinical factors were linked with ISR in previous studies, but little is known about the molecular mechanism of ISR [5]. Restenosis after PCI is considered a distinct pathophysiological process instead of an accelerated form of post-intervention atherosclerosis. Inflammatory cell adhesion, extracellular matrix deposition, hyperplasia and migration of endothelial and vascular smooth muscle cells, directly associated with injury to the vessel wall caused by stent implantation, are important factors contributing to restenosis after vascular intervention [6–8]. In addition, injured endothelial cells release more endothelin-1, angiotensin II, and growth factors (TGF-β,VEGF), but less nitric oxide (NO), which could further contribute to endomembrane hyperplasy and smooth muscle cell proliferation, leading to the gradual narrowing of ISR [9,10].

The effects of polymorphisms in genes encoding different enzymes, receptors, and growth factors on ISR or CAD are being actively investigated. Previously published reports have suggested a relationship between the occurrence of ISR or CAD and genetic variation occurring as single nucleotide polymorphisms (SNPs) in the endothelial nitric oxide synthase gene (*eNOS*) [11–13], the angiotensin converting enzyme gene (*ACE*) [14], the angiotensin II type 1 receptor gene (*AT1R*) [15,16], *TGF-β* [17–19], and *VEGF* [20,21]. However, the potential roles of functional polymorphisms in these genes in restenosis of DES have not been characterized. We hypothesized that genetic risk factors, that are associated with BMS-ISR or CAD may be associated with ISR after DES implantation. Thus the purpose of this study was to evaluate the associations between common SNPs occurring in the aforementioned genes and the risk of ISR in Chinese Han patients with stable CAD after DES implantation.

## Materials and Methods

### Ethics statement

Written informed consent was obtained from each patient. The study protocol conforms to the ethical guidelines of the 1975 Declaration of Helsinki and was approved by the Human Ethics Committee of Nanchang University Second Affiliated Hospital.

### Patient population

From January 2011 to September 2012, a total of 476 consecutive patients with a diagnosis of CAD and underwent successful target vessel revascularization with DES for the first time in Nanchang University Second Affiliated Hospital were respectively enrolled in a gene database. All patients had stent implantation for de novo lesions in native and non-grafted vessels and received regular followed up at least for 6 months. All patients had all treated lesions in one segment in our study. According to the clinical practice of Nanchang University Second Affiliated Hospital, an angiography was suggested to re-perform to assess the progress of coronary lesions and in-stent patency in 10–12 months after PCI. We recorded all patients who went back to hospital and performed repeated coronary angiography after discharge. Exclusion criteria were patients with evidence of a current infective inflammatory or neoplastic condition; acute myocardial infarction within the previous 4 weeks; previous PTCA with or without stent implantation in the same coronary artery; hepatic or renal dysfunction; disturbances of blood coagulation; rheumatologic disorders; tumor.

### Data collection and definition of risk factors

Information on demographic characteristics and other risk factors was collected by use of a structured questionnaire involving body mass index (BMI), the history of hypertension, diabetes, lifestyle (e.g., cigarette smoking and consumption of alcohol drinking), the family history of CAD and medications.

For risk factors, the following definitions were used: individuals were defined as hypertensive if their blood pressure was 140/90 mmHg or if they were receiving any antihypertensive treatment; individuals with a history of diabetes or those receiving any antidiabetic medication were considered to be diabetic; individuals with previous stroke were defined who had a history of stroke before entry into the study. The family history of CAD was considered positive for CAD if at least one first-degree male relative was diagnosed with CAD by the age of 55 or one first-degree female relative was diagnosed with CAD by the age of 65 years. Smoking was defined as the daily smoking of any number of cigarettes or cigars. Alcohol drinking in our study was defined as consumption of ≥1 drink/day (≥12.5 g/day of ethanol).

### Interventional procedure and angiography data

Each patient was preprocedurally administered with aspirin, 100 to 300 mg/d, and clopidogrel, 300 mg. Quantitative coronary angiography (QCA) analysis was performed by a validated, edge detection system. The DES were deployed according to the results of QCA. A successful PCI procedure was defined as a final residual stenosis less than 20% and Thrombolysis in Myocardial Infarction (TIMI) flow grade III. During angioplasty every patient was given an intra-arterial injection of 70 U/kg of heparin and intracoronary injection of 250 ug of nitroglycerine. ISR was defined as the narrowing of the vessel lumen by >50% within or up to 5 mm of the previously implanted stent, which was detected using coronary angiography. The cardiologists performing the QCA analyses were blinded to the results of genotyping analysis.

### Selection of SNPs and genotyping

The following SNPs were determined: eNOS gene G298A (rs#1799983) and T786C (rs#2070744), TGF-b gene C509T (rs#1800469), ACE gene I/D (rs#1799752), AT1R gene A1166C (rs#5186), VEGF gene C936T (rs#3025039) (Table 1). Candidate genes were selected based on review of the literature showing strong association with ISR and/or CAD. SNPs within each gene were also selected based on previous publication, focusing on functional SNPs and with a minor allele frequency of >5% in Chinese Han.

**Table 1:** Genomic characteristic of studied SNPs.

Gene	SNP	rs number	Chromosome	Major/minor	MAF	AA change
*eNOS*	G298A	rs1799983	7q35-36	G/A	0.111	Glu-ASP
*eNOS*	T786C	rs2070744	7q35-36	T/C	0.112	NA(5’UTR)
*TGF-β*	C509T	rs1800469	19q13	C/T	0.442	NA(near gene 5’)
*ACE*	I/D	rs1799752	17q23	I/D	0.482	NA(near gene 5’)
*AT1R*	A1166C	rs5186	3q24	A/C	0.053	NA(3’UTR)
*VEGF*	C936T	rs3025039	6p21	C/T	0.177	NA(3’UTR)

AA: amino acid; MAF: minor allele frequency; *eNOS*: endothelial nitric oxide synthase gene; *TGF-β*: transforming growth factor beta gene; *ACE*: angiotensin-converting enzyme gene; *AT1R*: angiotensin type 1 receptor gene; *VEGF*: vascular endothelial growth factor gene.

DNA samples were purified from peripheral blood leukocytes, which had been stored frozen at -20°C. Purification was performed using a DNA Purification kit (Promega Inc., Madison, WI, USA) according to the manufacturer's instructions. All primers were designed using a web tool (Beckman Coulter Inc., Fullerton, California, USA, http://www.autoprimer.com), synthesized, and detected by Invitrogen, Shanghai, China. For *ACE* ID polymorphism, we used a PCR-based method for genotyping the polymorphism, as previously described [22]. Genotyping was conducted by Orchid BioSciences using the GenomeLab SNPstream genotyping platform (Beckman Statistical analyses) and SNPstream software suite. Genotyping was performed by experienced staff blind to subject data.

### Statistical analyses

Continuous variables were presented as mean ± standard deviation (SD) or median and interquartile range (IQR) (depending on the normality of distribution). Variables were compared between the ISR group and non-ISR group using unpaired student’s t-tests for normally-distributed continuous variables or Wilcoxon rank sum tests for skewed variables. Categorical variables are represented by frequencies and percentages, and were compared using chi-squared tests. A chi-squared test was used to compare the observed numbers of each genotype with those expected for a population in Hardy-Weinberg equilibrium (HWE). Haploview version 4.2 (Daly Lab, Cambridge, MA, USA) was also used to determine the linkage disequilibrium (LD) block and haplotypes between two *eNOS* SNPs. Logistic regression was performed to assess the association between the presence of a particular genotype and the angiographic outcome. Additive, dominant, and recessive genetic models of the minor allele were assumed in association analyses, and analyses were performed with or without adjustment for confounding risk factors. All variables that resulted with a P value <0.25 in group comparison were entered into a multivariate model for ISR to test for independent effects.

All analyses were performed using SAS Version 9.1 (SAS Institute, Cary, North Carolina, USA). All statistical tests were based on a two-tailed probability and p<0.05 was considered statistically significant.

## Results

### Comparison of baseline clinical data between ISR and non-ISR groups

We excluded 51 patients who became lost to follow-up and thus did not receive coronary angiography to detect ISR after PCI. Finally, a total of 425 patients (mean age of 60.67±6.13 years, 111 women) with 485 lesions who underwent DES implantations were enrolled in this study. No significant differences were observed in clinical variables between the eligible patients and patients lost to follow up (S1 Table). Fifty four patients developed ISR during a median follow-up period of 12 months. Compared with the non-ISR group, patients with ISR had higher incidence of smoking (13.0% vs 4.9%, *p* = 0.02), longer length of stent per lesion (20.43±5.95 mm vs 17.43±5.44 mm, *p*<0.01), and smaller stent diameter (2.78±0.22 mm vs 2.89±0.25 mm, *p*<0.01). Age, sex distribution, BMI, drinking, diabetes mellitus, low-density lipoprotein (LDL) cholesterol, high-density lipoprotein (HDL) cholesterol, left ventricle ejection fraction (LVEF), target vessel location, number of stent in treated lesions, predilatation, postdilatation, incidence of hypertension, previous stroke, family history of CAD, time to angiography, and medications (antiplatelet therapy, statins, angiotensin converting enzyme inhibitor [ACEi]/angiotensin receptor blocker [ARB]), beta-blockers, nitrates, and proton pump inhibitors) did not differ between ISR and non-ISR groups (Table 2).

**Table 2:** Characteristics of patients included in the study.

Variable	ISR (n = 54)	non-ISR (n = 371)	*P*
Age (years)	61.37±6.0	60.56±6.15	0.36
Female	13(24.1)	98(26.4)	0.71
BMI (kg/m^2^)	23.34±1.64	23.70±1.62	0.13
Smoking	7(13.0)	18(4.9)	0.02
Drinking	14(25.9)	126(34.0)	0.24
Diabetes mellitus	20(37.0)	109(29.4)	0.25
Hypertension	44(81.5)	281(75.7)	0.35
Previous Stroke	2(3.7)	12(3.2)	0.86
Family History of CAD	12(22.2)	62(16.7)	0.32
LDL cholesterol (mmol/l)	3.02±0.60	2.98±0.52	0.59
HDL cholesterol (mmol/l)	1.32±0.26	1.37±0.28	0.27
LVEF (%)	59.48±5.47	60.55±5.26	0.17
Medications n (%)			
Antiplatelet therapy	54(100)	371(100)	1
Statins	52(96.3)	344(92.7)	0.33
ACEi/ARB	53(98.2)	340(91.6)	0.09
β-blockers	50(92.6)	344(92.7)	0.97
Nitrates	49(90.7)	314(84.6)	0.24
PPIs	8(14.8)	47(12.7)	0.66
Target vessel location n (%)			0.67
LM	1(1.85)	18(4.2)	
LAD/D1	25(46.3)	172(43.4)	
LCX/OM/IM	10(18.5)	77(20.8)	
RCA/PDA/PLA	18(33.3)	104(28.0)	
Nubmber of stent in treated lesions			0.25
One stent	44(81.5)	325(87.6)	
Two stents	10(18.5)	42(11.3)	
Three stents	0(0)	4(1.1)	
Length of stent per lesion (mm)	20.43±5.95	17.43±5.44	<0.01
Stent diameter (mm)	2.78±0.22	2.89±0.25	<0.01
Predilatation	21(38.9)	115(31.0)	0.26
Postdilatation	15(27.8)	94(25.3)	0.70
Median (IQR) time to QCA (month)	12(11–12)	12(11–12)	0.48

BMI: body mass index; CAD: coronary artery disease; LDL: low-density lipoproteins; HDL: high-density lipoproteins; LVEF: left ventricle ejection fraction; ACEi: ACE inhibitor; ARB: angiotensin receptor blocker; PPIs: proton pump inhibitors; LM: left main; LAD/D1: left anterior descending artery/1st diagonal branch; LCX/OM/IM: left circumflex artery/obtuse marginal branch/intermediate branch; RCA/PDA/PLA: right coronary artery/posterior descending artery/posterolateral artery; QCA: quantitative coronary angiography.

### Association of polymorphisms with ISR after DES implantation

The genotyping success rates of the six SNPs ranged from 99.8–100%. All SNPs genotyped conformed to HWE (p>0.05), minimizing the possibility of selection bias. Genotype and allele distributions of the six SNPs among the groups are shown in Table 3. There were no differences in genotype and allele distribution of the SNPs between the eligible patients and patients lost to follow up (S2 Table). Significant differences were obtained for *eNOS* T786C polymorphism between ISR group and non-ISR group. ISR was significantly more frequent in patients with the CC genotype of T786C polymorphism versus patients with the TC and TT genotypes (p = 0.03). There were more minor allele carriers of *eNOS* T786C polymorphism in the group of patients with ISR compared in the group without ISR (22.2% vs 12.7%, *p*<0.01). Restenosis frequency did not differ for individual genotypes and alleles of other analyzed polymorphisms. The frequencies of minor alleles of the analyzed polymorphisms were similar to those observed in 1000 Genomes Project (http://www.1000genomes.org//) among Chinese Han.

**Table 3:** Genotype and allele frequency.

SNP	Genotype and allele	ISR (n = 54)	Non-ISR (n = 371)	*P*
***eNOS* G298A**	GG	36(66.7)	278(75.1)	0.39
	GA	17(31.5)	85(23.0)	
	AA	1(1.8)	7(1.9)	
	G:A	0.82:0.18	0.87:0.13	0.24
***eNOS* T786C**	TT	33(61.1)	285(76.8)	0.03
	TC	18(33.3)	78(21.0)	
	CC	3(5.6)	8(2.2)	
	T:C	0.78:0.22	0.87:0.13	<0.01
***TGF-β* C509T**	CC	15(27.8)	108(29.1)	0.97
	CT	26(48.1)	178(48.0)	
	TT	13(24.1)	85(22.9)	
	C:T	0.52:0.48	0.53:0.47	0.88
***ACE* I/D**	II	15(27.8)	105(28.4)	0.96
	ID	28(51.9)	185(50.0)	
	DD	11(20.4)	80(21.6)	
	I:D	0.54:0.46	0.54:0.46	0.94
***AT1R* A1166C**	AA	45(83.3)	310(83.5)	0.86
	AC	9(13.2)	59(15.9)	
	CC	0(0)	2(0.5)	
	A:C	0.92:0.08	0.92:0.08	0.96
***VEGF* C936T**	TT	36(66.7)	256(69.0)	0.66
	TC	17(31.5)	101(27.2)	
	CC	1(1.8)	14(3.8)	
	T:C	0.82:0.18	0.83:0.17	0.95

SNP: single nucleotide polymorphism; ISR: in-stent restenosis; *eNOS*: endothelial nitric oxide synthase gene; *TGF-β*: transforming growth factor beta gene; *ACE*: angiotensin-converting enzyme gene; *AT1R*: angiotensin type 1 receptor gene; *VEGF*: vascular endothelial growth factor gene.

Of the six SNPs, only *eNOS* T786C polymorphism was associated with an increased risk of ISR in additive and dominant models with ORs of 1.903 (95% CI: 1.162–3.116, p = 0.01) and 2.109 (95% CI: 1.160–3.835, p = 0.01), respectively. When additionally adjusted for BMI, drinking, smoking, diabetes mellitus, LVEF, ACEi/ARB, nitrate, number of stent in treated lesions, length of stent per lesion, and stent diameter, the significance remained in the additive and dominant models with ORs of 1.870 (95% CI: 1.079–3.240, p = 0.03) and 2.045 (95% CI: 1.056–3.958, p = 0.03), respectively (Table 4). The overall pairwise LD constructed by the two *eNOS* SNPs are strong (*D’*:0.598, r^2^ = 0.357). Moreover, we observed no haplotype that significantly increased the risk of ISR.

**Table 4:** Association of polymorphisms with ISR after DES implantation.

Polymorphisms	Addictive model	*P*	Dominant model	*P*	Recessive model	*P*
	OR(95% CI)		OR(95% CI)		OR(95% CI)	
***eNOS* G298A**	**Per each A allele**		**(GA+AA) vs GG**		**AA vs (GA+GG)**	
Non-adjusted model	1.385(0.806–2.380)	0.24	1.511(0.819–2.789)	0.19	1.543(0.324–7.338)	0.59
Adjusted model	1.410(0.773–2.554)	0.26	1.589(0.07–3.129)	0.18	1.430(0.262–7.795)	0.68
***eNOS* T786C**	**Per each C allele**		**(TC+CC) vs TT**		**CC vs (TC+TT)**	
Non-adjusted model	1.903(1.162–3.116)	0.01	2.109(1.160–3.835)	0.01	2.669(0.686–10.389)	0.16
Adjusted model	1.870(1.079–3.240)	0.03	2.045(1.056–3.958)	0.03	2.579(0.579–11.480)	0.21
***TGF-β1* C509T**	**Per each T allele**		**(TC+TT) vs CC**		**TT vs (TC+CC)**	
Non-adjusted model	1.049(0.706–1.561)	0.81	1.068(0.565–2.017)	0.84	1.067(0.546–2.083)	0.85
Adjusted model	0.978(0.632–1.514)	0.92	0.859(0.432–1.708)	0.67	1.111(0.541–2.281)	0.77
***ACE* I/D**	**Per each D copy**		**(ID+DD) vs II**		**DD vs (ID+II)**	
Non-adjusted model	0.967(0.630–1.485)	0.88	1.04(0.550–1.965)	0.90	0.931(0.459–1.887)	0.84
Adjusted model	0.996(0.652–1.522)	0.99	1.215(0.616–2.395)	0.57	0.769(0.353–1.675)	0.51
***AT1R* A1166C**	**Per each C allele**		**(AC+CC) vs AA**		**CC vs (AC+AA)**	
Non-adjusted model	0.979(0.467–2.055)	0.96	1.016(0.472–2.188)	0.97	**—**	**—**
Adjusted model	1.198(0.535–2.680)	0.66	1.259(0.546–2.902)	0.59	**—**	**—**
***VEGF* C936T**	**Per each T allele**		**(CT+TT) vs CC**		**TT vs (TC+CC)**	
Non-adjusted model	1.014(0.602–1.708)	0.96	1.113(0.607–2.042)	0.73	0.481(0.062–3.734)	0.48
Adjusted model	1.053(0.602–1.841)	0.86	1.198(0.617–2.322)	0.59	0.455(0.056–3.689)	0.46

"**—**" not calculated because of low number of minor homozygotes

Adjusted models were additionally BMI, drinking, smoking, diabetes mellitus, LVEF, ACEi/ARB, nitrate, number of stent in treated lesions, length of stent per lesion, and stent diameter.

*eNOS*: endothelial nitric oxide synthase gene; *TGF-β*: transforming growth factor beta gene; *ACE*: angiotensin-converting enzyme gene; *AT1R*: angiotensin type 1 receptor gene; *VEGF*: vascular endothelial growth factor gene.

## Discussion

In this study, we investigated the association of six common genetic variants with the risk of ISR after DES implantation. We demonstrated that the C allele of the *eNOS* T786C polymorphism was linked with an increased risk of ISR in Chinese patients treated with DES. Multivariate analysis showed that this association was independent of other traditional risk factors of ISR.

Several studies have demonstrated positive correlations between the presence of the *eNOS* T786C mutant allele and the development of cardiovascular disease in Asian populations. Nakayama et al. found that the *eNOS* T786C polymorphism was linked to coronary artery spasm and myocardial infarction occurrence in Japanese [23,24]. Jo et al. and Kim et al. also demonstrated that the T786C polymorphism was associated with a predisposition to coronary artery disease and myocardial infarction among Koreans [25,26]. Similarly, Yi Han et al. reported that the T786C polymorphism of the *eNOS* appears to be an independent risk factor for coronary heart disease in Chinese Hans [27]. More recently, a large meta-analysis conducted by Himanshu Rai et al, involving a total sample size of 69,235 subjects, confirmed the association of the *eNOS* T786C polymorphism with the presence of coronary heart disease in different populations [28].

Although the effect of the *eNOS* T786C polymorphism on CAD has been investigated extensively, a specific relationship between the T786C polymorphism and responses to interventional therapy for CAD is a relatively new area of interest. Gomma et al. first assessed the relationship between the *eNOS* polymorphism and restenosis following coronary BMS deployment [11]. They found that the T786C and G298A polymorphisms modulated the response to BMS, and that the C allele of the T786C polymorphism was a strong predictor for ISR. Our current study supplies complementary evidence for an important role of *eNOS* in the pathomechanism of ISR after DES implantation. The association of the *eNOS* T786C polymorphism with ISR persisted after adjustment for other potential risk factors, indicating that the effect may not be mediated by known risk factors for ISR, but instead by genetic factors. This suggests that carriers of this particular genotype or allele of the *eNOS* T786C polymorphism may experience different pathophysiological effects after DES implementation. No association was observed between other potential genetic risk factors and ISR of DES in this study. We propose that the discrepancy in the results between studies could be due to the different genetic composition of populations, insufficient sample size, and different criteria of CAD patients in different studies.

Endothelial dysfunction is characterized by an impairment of endothelium-dependent relaxation due to decreased generation and bioavailability of nitric oxide (NO) in the vessel, and is a key event in the pathogenesis of in-stent restenosis. NO is generated in the endothelium during the conversion of L-arginine to L-citrulline in the presence of the nicotinamide adenine dinucleotide phosphate (NADPH)-dependent enzyme, eNOS [29]. The biological effects of endothelial-derived NO include vasodilation, inhibition of vascular smooth muscle cell (SMC) growth, anti-atherosclerotic properties, prevention of platelet aggregation, and inhibition of the adhesion of white cells to the blood vessel wall [30–32]. The T786C polymorphism was reported to decrease *eNOS* promoter activity by ~50% in human umbilical endothelial cells [24]. Miyamoto Y et al. reported that the activity of replication protein A1 (RPA1) reduces the transcriptional activity of *eNOS* with the 786C allele [33]. Additionally, serum nitrite/nitrate levels among individuals carrying the *eNOS* T786C mutation were significantly lower than among those without the mutation. Doshi et al. provided the first demonstration in human myocardial tissue that the T786C promoter polymorphism is associated with a significant reduction of *eNOS* mRNA expression and a corresponding reduction in eNOS protein [34]. Reduced or impaired synthesis of NO, due to the *eNOS* genetic polymorphism may promote vascular SMC proliferation and thus may induce neointimal hyperplasia, leading to coronary in-stent restenosis. However, ISR lesions in DES are more often hypocellular heterogeneous tissue comprised of scanty smooth muscle cells and abundant extracellular matrix materials such as proteoglycan and fibrin [35]. Endothelial disturbance may delay the vascular healing process, which in turn may contribute to excessive fibrin thrombus formation causing coronary flow disturbance and the development of ISR [36]. In addition, Nakazawa G et al. reported earlier and more frequent neoatherosclerosis within the restenostic tissue in ISR in patients treated with DES [37]. The T786C polymorphism of *eNOS* could modulate the effects of statin on anti-inflammatory effect and oxidative stress, which accelerated atherogenesis [38]. Collectively, genomic alterations in *eNOS* resulted in the deregulation of gene expression profiles and subsequently led to the development of ISR after DES implantation.

In this study, we analyzed the relationship between other potential risk loci and the risk of ISR. Among the *eNO*S polymorphisms, G298A has been extensively studied for a potential association with cardiovascular disease. The A allele of the G298A polymorohism has been reported as a risk factor for coronary in-stent restenosis [11,12]. The 298A variant has altered function, as it results in protein that is more susceptible to proteolytic cleavage, and thus leads to lower eNOS levels [39,40]. The influence of the *TGF-β1* C509T polymorphism on cardiovascular disease was also described in previous studies [17,41]. Recently, three *TGF-β1* polymorphisms (1800470, rs2285094, and rs6999447) were reported to be associated with ISR in patients treated with BMS, even after the adjustment for many risk factors [20]. The *ACE* I/D and *AT1R* A1166C polymorphisms are in genes that act at different steps of the renin-angiotensin system enzymatic cascade. Several studies have reported that individuals with the *ACE* DD genotype have a significantly higher risk for ISR and have more late loss and lower minimal lumen diameter measured by 6-month angiography [42,43]. The *AT1R* A116C polymorphism was also reported to confer risk of ISR after coronary stenting [15,16]. VEGF is a potent stimulator of endothelial cell migration and may be critically involved in the modulation of vascular healing. A recent study demonstrated that an increase of VEGF plasma levels was associated with restenosis of DES [44]. More recently, a *VEGF* plolymorphsim was linked to ISR of BMS in stable CAD patients [20]. In this study, we did not confirm these previous findings. The discrepancy in findings may be partly explained by the heterogeneity of the studies due to different inclusion and exclusion criteria or to different genetic and ethnic backgrounds.

This study has several limitations that should be acknowledged. First, this study is a single-center retrospective study and is therefore subject to the limitations of this type of clinical analysis. Thus, we are unable to confirm or refute causality, so large prospective studies will be needed to validate our results. Second, the study was limited to one specific population, namely Chinese Han, and considering the dramatic variation in the distribution of these genes with ethnicity, we cannot generalize our findings to other populations. Third, we have not directly measured vascular NO production and eNOS expression level in this study, we can infer that the presence of the C allele of the T786C variant exerts its effects on endothelial function.

In conclusion, this study demonstrated a novel association of *eNOS* variants with risk of ISR in Chinese patients treated with DES, which may have clinical implications. Our results provided some evidence warranting the inclusion of *eNOS* variants in future genotype-specific risk stratification for patients treated with DES. However, future well-designed large-scale studies are warranted.

## Supporting Information

S1 Dataset Genotyping results of patients. (XLS)

S1 Fig Typical images of ISR and non-ISR by coronary angiography. (A) Patient A with the *eNOS* 786 CC genotype had a diagnosis of stable coronary artery disease. A pre-PCI coronary angiogram demonstrated 95% stenosis in the mid portion of the left anterior descending artery (LAD). The PCI was performed with the placement of the stent in LAD. Follow-up coronary angiogram performed 11 months after PCI showed a significant in-stent restenosis (>50%) in LAD (arrow). (B) Patient B had the *eNOS* 786 TT genotype and a diagnosis of stable coronary artery disease. A pre-PCI coronary angiogram demonstrated 95% stenosis in the proximal portion of LAD. PCI was performed with the placement of stent in the LAD. Follow-up coronary angiogram performed 12 months after PCI showed no significant in-stent restenosis.
(TIF)

S1 Table Characteristics of eligible patients and patients lost to follow up. (DOCX)

S2 Table Genotype and allele frequency in eligible and patients lost to follow up. (DOCX)
