# Supplementary figures and images for: Association of the Endothelial Nitric Oxide Synthase Gene T786C Polymorphism with In-Stent Restenosis in Chinese Han Patients with Coronary Artery Disease Treated with Drug-Eluting Stent
Source: PLoS One. 2017 Jan 27;12(1):e0170964. doi: 10.1371/journal.pone.0170964 (PMC5271353; doi:10.1371/journal.pone.0170964)

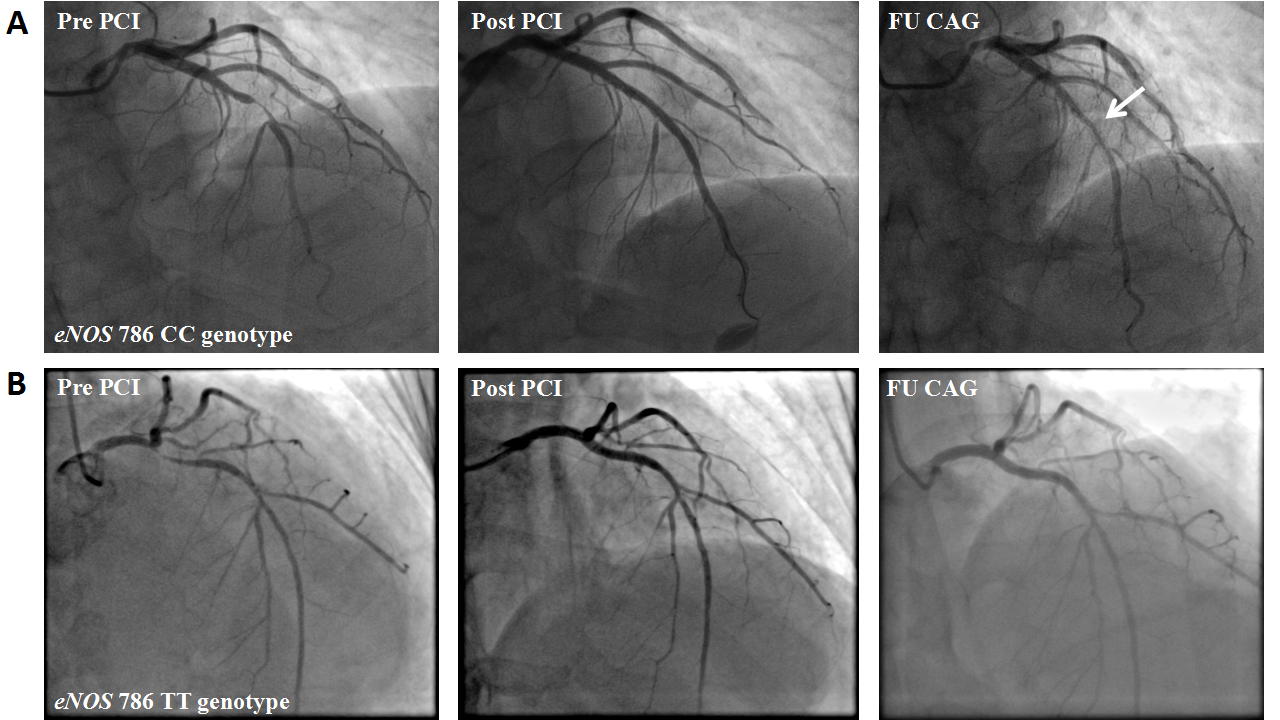

Supplement: S1 Fig — (A) Patient A with the eNOS 786 CC genotype had a diagnosis of stable coronary artery disease. A pre-PCI coronary angiogram demonstrated 95% stenosis in the mid portion of the left anterior descending artery (LAD). The PCI was performed with the placement of the stent in LAD. Follow-up coronary angiogram performed 11 months after PCI showed a significant in-stent restenosis (>50%) in LAD (arrow). (B) Patient B had the eNOS 786 TT genotype and a diagnosis of stable coronary artery disease. A pre-PCI coronary angiogram demonstrated 95% stenosis in the proximal portion of LAD. PCI was performed with the placement of stent in the LAD. Follow-up coronary angiogram performed 12 months after PCI showed no significant in-stent restenosis. (TIF) [file pone.0170964.s002.tif]
