# Supplementary material for: Association of the Endothelial Nitric Oxide Synthase Gene T786C Polymorphism with In-Stent Restenosis in Chinese Han Patients with Coronary Artery Disease Treated with Drug-Eluting Stent
Source: PLoS One. 2017 Jan 27;12(1):e0170964. doi: 10.1371/journal.pone.0170964 (PMC5271353; doi:10.1371/journal.pone.0170964)
Supplement: S1 Table — (DOCX) [file pone.0170964.s003.docx]

**S1 Table. Characteristics of eligible and excluded patients**

| **Variable** | **Eligible patients(n=425)** | **Excluded patients(n=51)** | ***P*** |
| --- | --- | --- | --- |
| Age (years) | 60.67±6.13 | 61.71±6.34 | 0.24 |
| Female | 111(26.1) | 15(29.4) | 0.61 |
| BMI (kg/m^2^) | 23.65±1.62 | 23.78±1.65 | 0.60 |
| Smoking | 25(5.9) | 2(3.9) | 0.56 |
| Drinking | 140(32.9) | 19(37.3) | 0.54 |
| Diabetes mellitus | 129(30.4) | 13(25.5) | 0.47 |
| Hypertension | 325(76.5) | 35(68.6) | 0.22 |
| Previous Stroke | 14(3.3) | 3(5.9) | 0.35 |
| Family History of CAD | 74(17.4) | 10(19.6) | 0.70 |
| LDL cholesterol (mmol/l) | 2.99±0.53 | 2.96±0.59 | 0.72 |
| HDL cholesterol (mmol/l) | 1.36±0.28 | 1.38±0.21 | 0.74 |
| LVEF (%) | 60.41±5.30 | 60.86±5.60 | 0.57 |
| Medications n (%) |  |  |  |
| Antiplatelet therapy | 425(100) | 51(100) | 1 |
| Statins | 396(93.6) | 48(94.1) | 0.80 |
| ACEi/ARB | 393(92.5) | 48(94.1) | 0.67 |
| β-blockers | 394(92.7) | 46(90.2) | 0.52 |
| Nitrates | 363(85.4) | 45(88.2) | 0.59 |
| PPIs | 55(12.9) | 6(11.8) | 0.81 |
| Target vessel location n (%) |  |  | 0.94 |
| LM | 19(4.5) | 3(5.9) |  |
| LAD/D1 | 197(46.4) | 24(47.1) |  |
| LCX/OM/IM | 87(20.5) | 9(17.7) |  |
| RCA/PDA/PLA | 122(28.7) | 15(29.4) |  |
| Nubmber of stent in treated lesions |  |  | 0.78 |
| One stent | 369(86.8) | 45(88.2) |  |
| Two stents | 52(12.2) | 6(11.8) |  |
| Three stents | 4(0.9) | 0(0) |  |
| Length of stent per lesion (mm) | 17.8±5.12 | 17.2±4.52 | 0.40 |
| Stent diameter (mm) | 2.87±0.25 | 2.92±0.21 | 0.23 |
| Predilatation | 136(32.0) | 17(33.3) | 0.85 |
| Postdilatation | 109(25.7) | 9(17.7) | 0.21 |
| Median (IQR) time to QCA (month) | 12(11-12) | — | — |

"**—**" not calculated because data were not available

BMI: body mass index; CAD: coronary artery disease; LDL: low-density lipoproteins; HDL: high-density lipoproteins; LVEF: left ventricle ejection fraction; ACEi: ACE inhibitor; ARB: angiotensin receptor blocker; PPIs: proton pump inhibitors; LM: left main; LAD/D1: left anterior descending artery/1st diagonal branch; LCX/OM/IM: left circumflex artery/obtuse marginal branch/intermediate branch; RCA/PDA/PLA: right coronary artery/posterior descending artery/posterolateral artery; QCA: quantitative coronary angiography
