# Supplementary material for: Association of the Endothelial Nitric Oxide Synthase Gene T786C Polymorphism with In-Stent Restenosis in Chinese Han Patients with Coronary Artery Disease Treated with Drug-Eluting Stent
Source: PLoS One. 2017 Jan 27;12(1):e0170964. doi: 10.1371/journal.pone.0170964 (PMC5271353; doi:10.1371/journal.pone.0170964)
Supplement: S2 Table — (DOCX) [file pone.0170964.s004.docx]

**S2 Table. Genotype and allele frequency in eligible and patients lost to follow up**

| **SNP** | **Genotype and allele** | **Eligible patients (n=425)** | **Excluded patients (n=51)** | ***P*** |
| --- | --- | --- | --- | --- |
| ***eNOS*  G298A** | GG | 314(74.1) | 39(76.5) | 0.93 |
|  | GA | 102(24.0) | 11(21.6) |  |
|  | AA | 8(1.9) | 1(2.0) |  |
|  | G:A | 0.86:0.14 | 0.87:0.13 | 0.75 |
| ***eNOS* T786C** | TT | 318(74.8) | 38(74.5) | 0.48 |
|  | TC | 96(22.6) | 13(25.5) |  |
|  | CC | 11(2.6) | 0(0) |  |
|  | T:C | 0.86:0.14 | 0.87:0.13 | 0.75 |
| ***TGF-β* C509T** | CC | 123(28.9) | 14(27.5) | 0.98 |
|  | CT | 204(48.0) | 25(49.0) |  |
|  | TT | 98(23.1) | 12(23.5) |  |
|  | C:T | 0.53:0.47 | 0.52:0.48 | 0.85 |
| ***ACE* I/D** | II | 120(28.3) | 13(25.4) | 0.89 |
|  | ID | 213(50.2) | 26(51.0) |  |
|  | DD | 91(21.5) | 12(23.6) |  |
|  | I:D | 0.53:0.47 | 0.51:0.49 | 0.64 |
| ***AT1R* A1166C** | AA | 355(83.5) | 42(82.4) | 0.85 |
|  | AC | 68(16.0) | 9(17.6) |  |
|  | CC | 2(0.5) | 0(0) |  |
|  | A:C | 0.92:0.08 | 0.91:0.09 | 0.90 |
| ***VEGF* C936T** | TT | 292(68.7) | 36(70.6) | 0.84 |
|  | TC | 118(27.8) | 14(27.4) |  |
|  | CC | 15(3.5) | 1(2.0) |  |
|  | T:C | 0.83:0.17 | 0.84:0.16 | 0.66 |

SNP: single nucleotide polymorphism; ISR: in-stent restenosis; *eNOS*: endothelial nitric oxide synthase gene; *TGF-β*: transforming growth factor beta gene; *ACE*: angiotensin-converting enzyme gene; *AT1R*: angiotensin type 1 receptor gene; *VEGF*: vascular endothelial growth factor gene.
